# Mental health consequences of COVID-19 media coverage: the need for effective crisis communication practices

**DOI:** 10.1186/s12992-020-00654-4

**Published:** 2021-01-05

**Authors:** Zhaohui Su, Dean McDonnell, Jun Wen, Metin Kozak, Jaffar Abbas, Sabina Šegalo, Xiaoshan Li, Junaid Ahmad, Ali Cheshmehzangi, Yuyang Cai, Ling Yang, Yu-Tao Xiang

**Affiliations:** 1 Center on Smart and Connected Health Technologies, Mays Cancer Center, School of Nursing, UT Health San Antonio, 7703 Floyd Curl Drive, San Antonio, TX 78229 USA; 2 Department of Humanities, Institute of Technology Carlow, Carlow, Ireland R93 V960; 3 School of Business and Law, Edith Cowan University, Perth, WA 6027 Australia; 4 School of Tourism, Dokuz Eylül University, 35680 Foça, İzmir, Turkey; 5 Antai College of Economics and Management, and School of Media and Communication, Shanghai Jiao Tong University, Shanghai, 200240 China; 6 Department of Microbiology, Faculty of Medicine, University of Sarajevo, 71000 Sarajevo, Bosnia and Herzegovina; 7 Program of Public Relations and Advertising, Beijing Normal University-Hong Kong Baptist University United International College, Zhuhai, Guangdong China; 8 Prime Institute of Public Health, Peshawar Medical College, Warsak Road, Peshawar, 25160 Pakistan; 9 Head of Department of Architecture and Built Environment; Professor of Architecture and Urban Design, Faculty of Science and Engineering, University of Nottingham Ningbo China, Ningbo, Zhejiang, 315100 China; 10 The Network for Education and Research on Peace and Sustainability (NERPS), Hiroshima University, Hiroshima, Japan; 11 School of Public Health, Shanghai Jiao Tong University School of Medicine, Shanghai, China; 12 China Institute for Urban Governance, Shanghai Jiao Tong University, Shanghai, China; 13 Department of Geriatrics, Xinhua Hospital, Shanghai Jiao Tong University School of Medicine, Shanghai, 200025 China; 14 Unit of Psychiatry, Institute of Translational Medicine, Faculty of Health Sciences; & Center for Cognition and Brain Sciences, University of Macau, Macao SAR, China

**Keywords:** COVID-19, Coronavirus, Mental health, Crisis communication, Infodemic, Misinformation, Disinformation

## Abstract

During global pandemics, such as coronavirus disease 2019 (COVID-19), crisis communication is indispensable in dispelling fears, uncertainty, and unifying individuals worldwide in a collective fight against health threats. Inadequate crisis communication can bring dire personal and economic consequences. Mounting research shows that seemingly endless newsfeeds related to COVID-19 infection and death rates could considerably increase the risk of mental health problems. Unfortunately, media reports that include infodemics regarding the influence of COVID-19 on mental health may be a source of the adverse psychological effects on individuals. Owing partially to insufficient crisis communication practices, media and news organizations across the globe have played minimal roles in battling COVID-19 infodemics. Common refrains include raging QAnon conspiracies, a false and misleading “Chinese virus” narrative, and the use of disinfectants to “cure” COVID-19. With the potential to deteriorate mental health, infodemics fueled by a kaleidoscopic range of misinformation can be dangerous. Unfortunately, there is a shortage of research on how to improve crisis communication across media and news organization channels. This paper identifies ways that legacy media reports on COVID-19 and how social media-based infodemics can result in mental health concerns. This paper discusses possible crisis communication solutions that media and news organizations can adopt to mitigate the negative influences of COVID-19 related news on mental health. Emphasizing the need for global media entities to forge a fact-based, person-centered, and collaborative response to COVID-19 reporting, this paper encourages media resources to focus on the core issue of how to slow or stop COVID-19 transmission effectively.

## Background

Similar to pandemics like the 1918–1919 influenza outbreak, the Coronavirus Disease 2019 (COVID-19) is a once-in-a-century event [1]. Different from previous global health crises, the impact of COVID-19 is not distant, rather, it is close to home, catastrophic, and ongoing—as of December 1st, approximately 63.3 million confirmed cases and 1.47 million deaths were known to be caused by COVID-19 [2]. The scope and severity of the pandemic have further fueled a global mental health crisis, especially among underserved populations like older adults, healthcare professionals, and women [3]. It is estimated that in October 2020, more people in Japan have died of suicide (2153) than COVID-19 (2087) [4]. Compared to numbers in 2019, there was a 82.6% rise among Japanese women who died of suicide in October, 2020 [4].

Though almost a year has passed since the first COVID-19 outbreak, epidemiologists are still working on understanding COVID-19’s clinical features [5]. In addition to its unknown viral characteristics, a key contributor fueling the destructive power of COVID-19 is its unprecedented transmissibility [6–8]. COVID-19’s ability to spread fast and far in a short period is rare, even among other pandemics [6–8]. This rapid pace of transmission, coupled with consequent spikes in infection and death, has caused a range of physical and psychological issues in individuals across the globe [9]. Challenging to identify or fully “cure”, mental health services were facing numerous, but resource-constraining pandemics like COVID-19 have exacerbated these issues [9–12].

Mental health is “a state of well-being in which the individual realizes his or her own abilities, can cope with the normal stresses of life, can work productively and fruitfully, and is able to make a contribution to his or her community” [13]. Amid a global crisis, mental health issues can have severe health consequences on personal and population health, ranging from anxiety, distress or depression, to suicidal ideation or suicide [3, 14, 15]. COVID-19 has been a source of complex, multifaceted stress for many [16–22]. The fears and uncertainty associated with the virus, together with the anxiety and stress following from lockdowns and social distancing mandates, have exacerbated mental health issues to varying degrees throughout society [23–25]. Not only diminishing the mental health and well-being of individuals, COVID-19 has also limited the services people can access; the rationing of medical resources during the COVID-19 pandemic has instigated a restructuring and repurposing across mental health institutions to deal with the pandemic [26–28]. Well-intentioned measures, such as lockdowns and social distancing, have further diminished access to mental health services [10], with many providers forced to close; leaving people little to no access to on-site assistance [26–28].

In addition to (1) the fear and uncertainty associated with COVID-19, (2) the anxiety and distress caused by lockdowns and social distancing mandates, and (3) limited access to mental health services [23–25], the unending barrage of news from legacy media outlets and social media platforms has further complicated the situation [18, 29, 30]. Media attention has disproportionately directed toward the COVID-19 infodemic, with little consideration for how pandemic-related media coverage might influence people’s mental health. Moreover, the misinformation and disinformation surrounding COVID-19 - ranging from a false and misleading “Chinese virus” narrative to using disinfectants to “cure” COVID-19 - has affected individuals’ mental and physical health and well-being [18, 19, 29, 31, 32]. Although some useful insight is available, scarce research has explored ways to mitigate the mental health consequences of COVID-19 media coverage.

Evidence shows that in times of global crisis such as COVID-19, crisis communication can, cost-effectively, address multifaceted issues. Crisis communication refers to “the collection, processing, and dissemination of information required to address a crisis situation” [33]. Though many developments of the field of crisis communication occurred in the past decades (e.g., the situational crisis communication theory developed by Timothy Coombs in 1995), crisis communication has a long history and is often contributed to eminent public figures such as Caesar and Confucius [34–37]. With the help of exemplar (e.g., Johnson & Johnson’s effective management of the Cyanide-Laced Tylenol Capsules crisis), as well as inadequate crisis communication practices (e.g., the United States government’s mismanagement of Hurricane Katrina), a growing body of work has acknowledged crisis communication’s role in mitigate negative impacts of adverse events [38–40]. Therefore, to address this research gap, this paper aims to identify areas where legacy media reports on COVID-19 and social media-fueled infodemics can harm people’s mental health. This paper outlines potential crisis communication solutions that media and news organizations can adopt to alleviate the mental health consequences of COVID-19 coverage.

### Coverage of COVID-19 by legacy media

Legacy media encompasses “media originally distributed using a pre-internet medium (print, radio, television), and media companies whose original business was in pre-Internet media, regardless of how much of their content is now available online” [41]. Three forms of coverage can broadly classify the impact of legacy media coverage of COVID-19 on people’s mental health issues: (1) balanced, fact-based, and truth-oriented; (2) biased and misleading; and (3) false and dishonest.

#### Balanced, fact-based, and truth-oriented COVID-19 media coverage

COVID-19 media coverage is inherently harmful; the disease represents an ongoing, deadly pandemic [2]. This intrinsic negativity, which naturally transfers to media coverage of the virus, could cause mental health issues [42]. Research on media effects has long documented that negative news can lead to mild to severe mental health issues among consumers [42]. Importantly, due to the scale and severity of COVID-19, media attention has been disproportionately focused on pandemic-related news, which could further affect individuals already facing more significant mental health challenges [42]. It is important to note that while balanced, fact-based, and truth-oriented COVID-19 media coverage might be difficult to achieve, it is important that media organizations, as pillars of the Fourth Estate [43], strive to meet these standards to their best abilities.

#### Biased and misleading COVID-19 media coverage

When news is biased and misleading, the adverse effects of COVID-19 media coverage on personal and population health and well-being could be more pronounced [44–46]. Previous studies found that right-leaning media outlets often issue biased and misleading reports on COVID-19 [46], which could, in turn, facilitate the spread of misinformation on the virus. Analysis of a sample of 38 million media reports from January 1 to May 25, 2020 shows that a staggering of 84% of misinformation distributed by legacy media was neither challenged or fact-checked before they reached the public, effectively exposing countless number of people to misinformation, such as “miracle cures” or the “Democratic Party hoax,” that could result in substantial human and economic consequences [47]. It is also important to note that fear and panic generated by COVID-19 related misinformation could have a long-lasting effect on people’s mental health that outlives COVID-19 media cycles [48].

#### False and dishonest COVID-19 media coverage

Perhaps the most problematic type of media coverage on COVID-19 involves content that is false and dishonest [18–21]. While legacy media practitioners uphold the founding pillars of the industry, journalistic values and ethical standards, the prevalence of narratives referring to the “Wuhan virus,” “Chinese virus,” and “China virus” in legacy media reports on COVID-19 suggests that some outlets are fully capable of producing baseless, and sensational news [18–21]. Directly associating a group of people, nation, and entire race to a virus will inevitably evoke substantial mental health concerns among those targeted [18–21].

Another irreversible negative effect of legacy media’s instigation of “fake news” is the deterioration of public trust around COVID-19 [49]. It is challenging to predict what might happen if people decide to ignore COVID-19 information disseminated through legacy media outlets, where health experts and government officials share the latest developments related to the virus. What is not difficult to imagine is the human and economic consequences tied to a deliberately “ignorant” public; the results could be catastrophic [50].

### COVID-19 infodemics and social media

COVID-19 infodemics are growing at a pandemic rate [51]. Infodemics involve the purposeful spread of misinformation and disinformation via the media, particularly on social media platforms. COVID-19 infodemics can detract from health experts’ efforts, fuelling public fear, uncertainty, and mistrust, which could have grave personal and economic consequences [51–56]. Infodemics involve an array of topics on which misinformation and disinformation are publicized through tweets and Facebook posts, oftentimes powered by interested individuals or groups with ulterior political and economic interests [55, 57]. Typical slants include QAnon conspiracies, the aforementioned “Chinese virus” narrative, and promoting the use of disinfectants to “cure” COVID-19 [51–56].

Not all COVID-19 infodemics are created equal [58]. For example, the infodemic that promoted the ingestion of disinfectant to utilize its “health benefits” had direct physical and mental health implications to a number of individuals [31, 32, 58, 59]. Between May 1st and June 30th, 2020, there were 15 reported cases of methanol poisoning due to drinking disinfectant; of these cases, four individuals died, and three were discharged with visual impairment [59]. Still, others may mistakenly trust U.S. leaders’ “sarcastic” remarks on COVID-19, which are repeatedly aired on legacy media and various other social media outlets [60, 61].

Resource constraints are a hallmark of COVID-19, and media resources are no exception. COVID-19 infodemics, along with smear campaigns endorsed by traditional media outlets, are an outrageous waste of public resources—global media attention should be focused on the health and well-being of the public, mainly because the pandemic is ongoing. In times of global crisis, media resources require investment in the issue of the day: how to slow or stop the spread of COVID-19 [62]. Considering the prevalence of misinformation and disinformation on legacy media and social media platforms, interventions are urgently needed to dispel COVID-19 infodemics and ensure related media coverage does not lead to unintended consequences; effective crisis communication practices are one such approach [62–64].

### Crisis communication amid COVID-19

In times of global pandemics such as COVID-19, crisis communication is indispensable in dispelling fear and uncertainty and unifying citizens in a collective fight against disease [62–64]. A fundamental attribution of crisis communication is that it is usually adopted as an emergency communication strategy when at least three crises are at play: (1) a crisis or unprecedented event with widespread personal and economic consequences (e.g., the COVID-19 pandemic); (2) a communication crisis that could prevent key stakeholders from working towards a solution (e.g., COVID-19 infodemics); and (3) a potential trust crisis either already present or in development, partially due to the first two crises (e.g., public trust crises).

To address these triple crises, society at large must take several steps: (1) rapidly develop an evidence-based, tailored disaster preparedness plan with the potential to curb the pandemic; (2) carefully execute this plan with speed and precision; and (3) communicate this plan and corresponding procedures effectively to the public in a timely, transparent, and truth-oriented fashion (i.e., effective crisis communication). Overall, effectively sharing public health updates with society in a reasonable and honest manner is paramount.

**Fig. 1:**
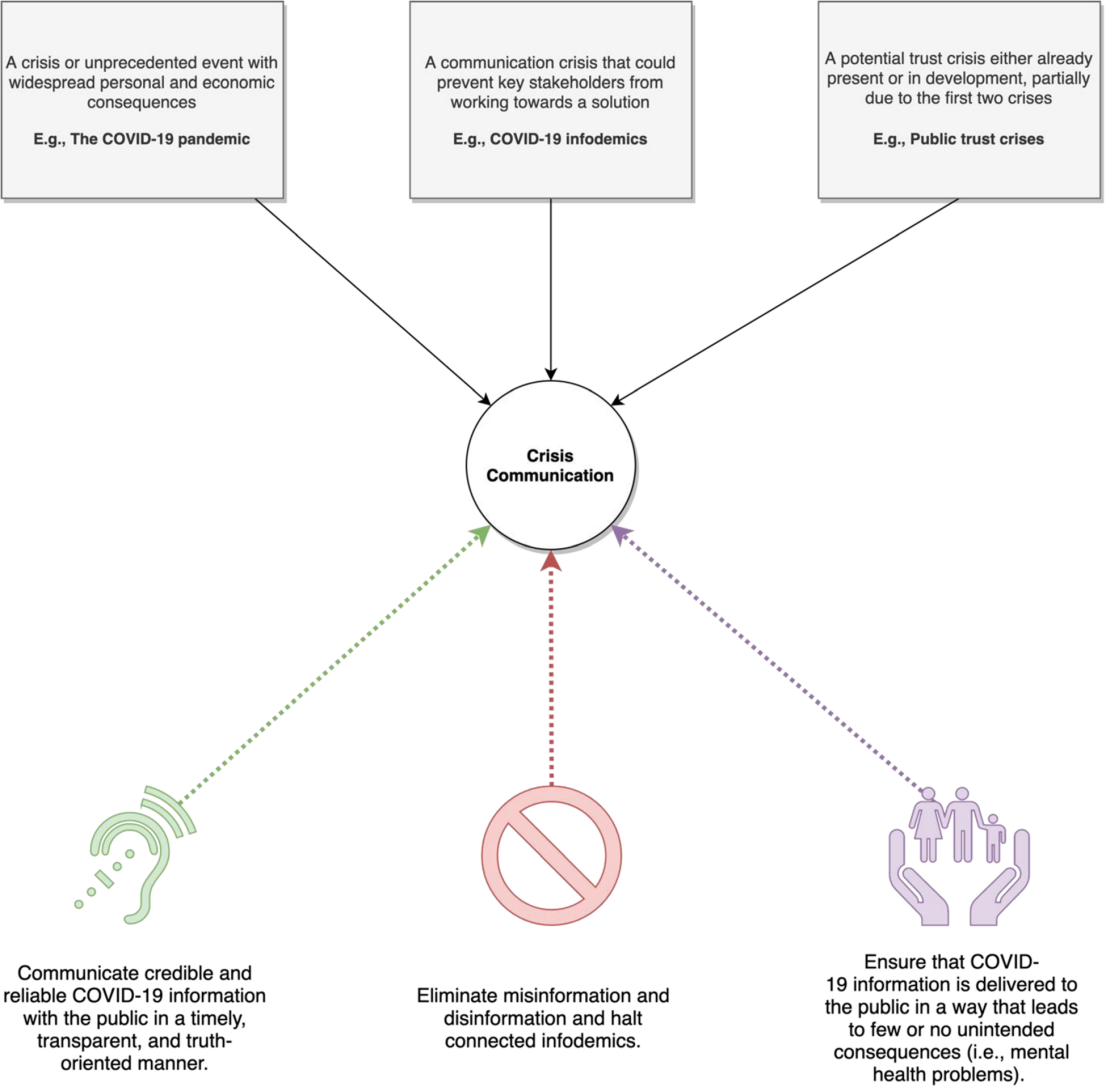
Antecedents to crisis communication and possible solutions

In addition to providing the public with trustworthy information, proactive decisions are needed from media professionals, health experts, and government officials to ensure effective delivery of COVID-19 updates to the public (i.e., so as not to cause unintended consequences involving mental health). In other words, crisis communication during COVID-19, especially in light of the mental health consequences associated with relevant media coverage, should have three objectives: (1) to communicate credible and reliable COVID-19 information with the public in a timely, transparent, and truth-oriented manner; (2) to eliminate misinformation and disinformation and halt connected infodemics; and (3) to ensure that the delivery of COVID-19 information to the public leads to no unintended consequences (i.e., mental health problems) (see Fig. 1).

### Communicate credible and reliable COVID-19 related information

During the pandemic, many governments, such as the Chinese [65], Irish [66], Finnish [67], and Norwegien government [68], have managed to communicate COVID-19 strategies effectively with the public. Take the Chinese government for instance. Starting from the first outbreak, the Chinese government has been delivering timely COVID-19 updates that are (1) tailored to the general public’s needs and wants to enhance relevancy; (2) disseminated via traditional and social media outlets to increase reach and impact; and (3) presented by key health and government officials to boost message credibility are available to the public daily [69–71]. Along with avoiding potential mental health issues, these crisis communication efforts also have the potential to dispel people’s fear and uncertainty about COVID-19 and improve their compliance with pandemic-related health and safety procedures such as lockdowns and face mask mandates [69–71].

Unprecedented times call for unprecedented measures [30]. Technology companies, including Google, Twitter, Facebook, and TikTok, can disseminate credible and reliable COVID-19 information by developing tailored algorithms to promote search results, tweets, or posts written by vetted epidemiologists or other health experts. Doing so could initiate a movement to communicate credible, reliable COVID-19 information with the public in a timely, transparent, and truth-focused fashion. Notably, the way public-facing messages are designed, developed, and delivered (i.e., in a persuasive manner that is relatable to the public) also influences communication outcomes [72].

### Eliminating COVID-19 infodemics

Relying on Health organizations and government agencies alone is not enough; all key stakeholders must be involved [69–71, 73]. Public health campaigns that target the dangers of COVID-19 infodemics require development, and information that educates individuals on how to avoid being a conduit of misinformation or disinformation is needed. Given that a considerable proportion of the public lack the health literacy needed to distinguish credible information from misinformation or disinformation [50], educational programs should be established to ensure that infodemics will become less prevalent both during COVID-19 and in the future.

Despite promising initiatives [74], media companies should assume a more significant role in controlling the spread of COVID-19 infodemics. Research shows that merely adding an accuracy reminder while people are perusing information online can substantially enhance their ability to identify fake news [75]. This finding is encouraging, as it suggests that effective measures to curb the spread of COVID-19 infodemics can be highly cost-effective. In addition to making individual decisions, perhaps social media companies should organize a collaborative response, such as through a crowdsourced and widely shared “Infodemic Response Checklist” [53]. This effort would help the social media environment at large establish a better system to protect the public from the harm of COVID-19 infodemics.

Overall, health experts should lead in quelling COVID-19 infodemics. As top epidemiologists like Dr. Anthony Fauci have demonstrated, health experts need to be closely connected with their main “customers” or the general public to facilitate effective communication [76–78]. Health experts also need to be more participatory in the public health decision-making process; in so doing, less disinformation will be disseminated by government officials while more decisions will be grounded in scientific evidence.

### Fact-based and people-centered COVID-19 crisis communication strategy

COVID-19 affects people of all demographics [79]. It is difficult not to form an opinion about an enduring pandemic that continues to threaten lives, livelihoods, and gross domestic product (GDP) [2]. However, given the personal and economic consequences tied to biased and misleading [44–46] or blatantly false and malicious [59–61] information, it is imperative for media professionals, health experts, and government officials to develop a fact-based, people-centered [17] COVID-19 crisis communication strategy. In the context of our study, fact-based and people-centered crisis communication strategy is defined as communication endeavors deliver facts that matter to the people without framing the numbers or statistics based on personal views or ulterior motives (e.g., political gains or economic interests).

This way, well-intentioned information can be effectively delivered to the public without unintended consequences. It is important to note that educational interventions might be also needed for healthcare professionals, as a growing body of research shows that healthcare professionals often lack necessary levels of knowledge or risk perception needed to be vigilant about COVID-19 misinformation or disinformation [80–82]. Considering the important role healthcare professionals serve in patient education and the fact that many healthcare professionals also face substantial mental health challenges [83], educational interventions may be incremental in addressing infodemic-induced challenges these frontline workers face.

### Concluding remarks

Overall, in times of global pandemics like COVID-19, crisis communication can play a key part in reducing fear and uncertainty while inspiring a unified fight against health threats [62–64, 84]. There has yet to be a national solution or unilateral communication during a pandemic, but considering the pronounced need for valuable media resources during COVID-19 for the greater good [50], health experts and media professionals have a responsibility to step up and put a stop to infodemics and smear campaigns. Stakeholders can battle inaccurate reporting with credible, reliable, and trustworthy information alongside well-developed tools and techniques in crisis communication. Transparency and legitimacy will ultimately help preserve people’s health and well-being while bringing global media attention back to a genuine public health concern: how to prevent COVID-19 from spreading.

For future research directions, we believe there is a pronounced need to capitalize on media or communication resources to develop timely health solutions that have the potential to avoid immediate human consequences caused by COVID-19. Since the onset of the pandemic, in Turkey alone, approximately 100 musicians have committed suicide due to financial problems caused by COVID-19 [85]. We believe regional, national, and international health organizations and government agencies should invest more media resources into informing and emphasizing help and resources available to people amid the pandemic, compared with updates on COVID-19 infection and death tallies. In other words, it is important for media organizations to honor their roles as pillars of the Fourth Estate amid COVID-19 [43], starting by pouring media resources into issues that matter to individuals’ lives and livelihoods, rather than sensational reports that might boost Nielsen ratings, increase sales numbers, fuel infodemics, yet add limited benefits to public health and welfare [47].

## Data Availability

No.

## Abbreviations

COVID-19: Coronavirus disease 2019
GDP: Gross domestic product
